# Glutaric Aciduria Presenting With an Acute Encephalitic Crisis: A Case Report

**DOI:** 10.7759/cureus.65722

**Published:** 2024-07-30

**Authors:** Manojkumar G Patil, Neha Tyagi, Om Prasanth Reddy Avuthu, Shradha Salunkhe

**Affiliations:** 1 Pediatrics, Dr. D. Y. Patil Medical College, Hospital and Research Centre, Dr. D. Y. Patil Vidyapeeth (Deemed to be University), Pune, IND

**Keywords:** pediatrics, amino acid, tandem mass spectrometry, neurology and critical care, genetic metabolic disorders

## Abstract

Glutaric aciduria type 1 (GA1) is an organic aciduria inherited in an autosomal recessive pattern, with an occurrence rate of one in 100,000. It is caused by a deficiency of the enzyme glutaryl-CoA dehydrogenase (GCDH), encoded by the *GCDH* gene on chromosome 19. It is an important enzyme in the catabolism of amino acids such as tryptophan, lysine, and hydroxylysine. Its deficiency leads to the accumulation of organic acids such as glutaric acid and 3-hydroxyglutaric acid, which interfere with cerebral energy metabolism and cause neurological symptoms. Here, we discuss the case of a six-month-old male child who presented with status epilepticus following an eight-day history of fever. The child was started on anti-epileptics. Initially, the child was on non-invasive ventilation and was later intubated and taken on a mechanical ventilator. A magnetic resonance imaging (MRI) scan of the brain was performed, and the findings suggested GA1. The child was started on carnitine after samples were sent for tandem mass spectrometry (TMS) and urine gas chromatography-mass spectrometry (GC/MS), which came out to be positive for GA1. Despite the timely intervention, the child did not survive. Most cases exhibit movement disorders, with many presenting in acute encephalitic crises. Additionally, a significant portion of patients experience an insidious onset of the disease. An MRI of the brain shows widened Sylvian fissures in the majority of cases. Treatment of GA1 includes dietary modifications, including a low-lysine diet and administering carnitine. Early diagnosis and management result in decreased mortality and morbidity, which underscores the need for newborn screening.

## Introduction

Glutaric aciduria refers to a group of organic acidurias inherited in an autosomal recessive pattern. There are three types, each caused by distinct genetic mutations affecting different enzymes. Glutaric aciduria type 1 (GA1), first described in 1975, has a prevalence of one in 100,000 [1]. Glutaric aciduria is often called cerebral organic aciduria due to its primarily neurological symptoms. GA1 is due to a deficiency of glutaryl-CoA dehydrogenase (GCDH), an enzyme within the acyl-CoA dehydrogenase family critical in the metabolism of amino acids such as tryptophan, lysine, and hydroxylysine. Its deficiency leads to elevated excretion of organic acids such as glutaric acid, 3-hydroxyglutaric acid (3-OH-GA), and glutaconic acid in the urine, as well as increased levels of glutarylcarnitine (C5DC) in plasma. The *GCDH* gene encoding the enzyme is located on chromosome 19p13.2 [2]. The majority of cases present between 2 and 37 months of age with movement disorders and acute encephalitis crises, often precipitated by intercurrent illness, infection, fasting, or immunization. In this report, we discuss the case of GA1 in a six-month-old child who presented with acute encephalitis.

## Case presentation

A six-month-old male child presented in a state of status epilepticus, exhibiting irritability between seizure-free periods. All seizure episodes had similar semiology, characterized by clonic posturing of the bilateral upper limbs, upward rolling of the eyes, and head deviation to the right, followed by postictal drowsiness and intermittent irritability. The child had a history of fever and cough for eight days, for which he was treated at a local clinic with symptomatic management. Seizures began eight days after the onset of the fever, prompting a visit to another hospital, where an EEG was performed but returned normal results.

Subsequently, the child was referred to our hospital. Upon admission, the child continued to seize with less than 10-second intervals between seizures. We administered IV levetiracetam at 60 mg/kg/day, IV fosphenytoin, and 3% NaCl. The child was born to third-degree consanguineous parents. An antenatal scan indicated uteroplacental insufficiency, but there was no history of maternal pregnancy-induced hypertension (PIH) or other comorbidities. At birth, the child did not require resuscitation but was admitted to the neonatal intensive care unit (ICU) for one day due to transient tachypnea. Metabolic disorder screening was not performed in the neonatal period. The child had also received prelacteal feeding in the form of honey.

Further history and examination revealed motor developmental delay, with other developmental domains appropriate for age. The child had only received birth immunizations as per the National Immunization Schedule and had been started on a family pot diet complementary to breastfeeding. Upon admission to the pediatric ICU, the child was febrile, tachycardic, and tachypneic, with an inspiratory stridor. No murmurs were heard on auscultation. Hepatomegaly was present, with the liver palpable 4 cm below the subcostal margin and a liver span of 13 cm. The child was placed on non-invasive ventilation due to increasing distress and a declining Glasgow Coma Scale (GCS) score. A lumbar puncture was conducted, and the cerebrospinal fluid (CSF) analysis returned normal results, including negative findings for virology (Table 1).

**Table 1:** Normal CSF report CSF: cerebrospinal fluid

CSF	Observed Values	Normal Values
Appearance	Clear	Clear
Leukocytes	3/mm^3^	<5/mm^3^
Protein	36 mg/dL	20–45 mg/dL
Glucose	80 mg/dL (corresponding blood sugar: 100 mg/dL)	75% of blood glucose

**Figure 1:**
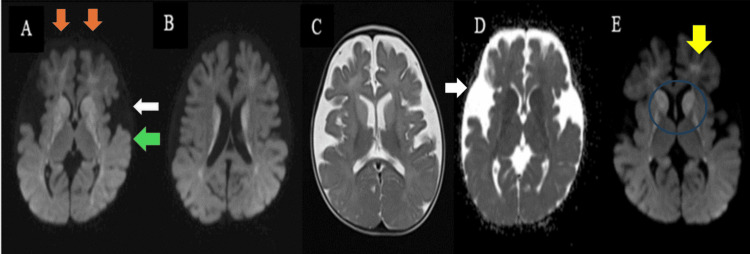
MRI brain axial images (A-D) Bifrontal (indicated by orange arrows) and temporal atrophy are noted (indicated by the green arrow), with prominent sulcal spaces and marked prominence of Sylvian fissures showing bat-wing appearance (indicated by white arrows). (E) A diffuse hyperintense signal is noted in bilateral lentiform nuclei, the head of the caudate nucleus (in blue circle), and bilateral frontal and peritrigonal white matter (indicated by the yellow arrow) on the FLAIR image MRI: magnetic resonance imaging; FLAIR: fluid-attenuated inversion recovery

The MRI findings suggested a metabolic disorder, possibly GA1 (Figure 1).

**Figure 2:**
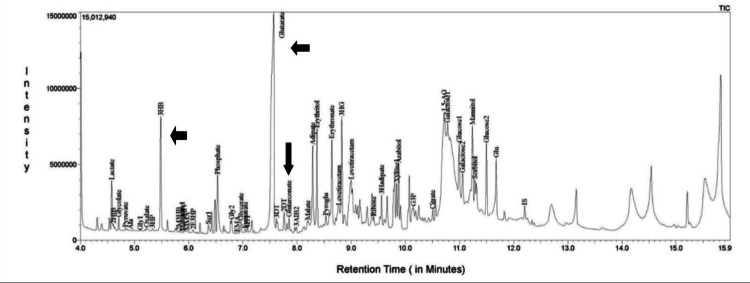
Total ion chromatogram of urinary metabolites by GC/MS showing increased GA, 3-OH-GA, and glutaconic acid GC/MS: gas chromatography-mass spectrometry; 3-OH-GA: 3-hydroxyglutaric acid

To confirm the diagnosis, we sent samples for tandem mass spectrometry (TMS) and urine gas chromatography-mass spectrometry (GC/MS). While awaiting results, we started the child on a metabolic cocktail, including L-carnitine (100 mg/kg/day), riboflavin (100 mg/day), biotin (10 mg/kg/day), vitamin B12 (1 mg/kg/day), thiamine (10 mg/kg/day), pyridoxine (50 mg/day), and folinic acid (20 mg/day). GC/MS results were positive, indicating elevated GA, 3-OH-GA, and glutaconic acid, suggesting the presence of GA1 (Table 2) (Figure 2).

**Table 2:** GC/MS report showing increased excretion of GA, glutaconic acid, and 3-OH-GA in urine, suggestive of GA1 GA: glutaric aciduria; 3-OH-GA: 3-hydroxyglutaric acid; GC/MS: gas chromatography-mass spectrometry

Metabolite Name	Observed Value	Control Value
GA	17.600	0.619
Glutaconic acid	0.440	0.001
3-OH-GA	3.802	0.001

Despite our efforts, the child's condition deteriorated, necessitating mechanical ventilation followed by high-frequency oscillatory ventilation. Hemodynamically, the child required inotropic support and continuous glucose monitoring due to recurrent hypoglycemia. Despite the intensive efforts of the entire unit, the child did not survive.

## Discussion

GCDH is an important enzyme in the catabolism of lysine, hydroxylysine, and tryptophan. It catalyzes the oxidation of glutaryl-CoA to glutaconyl-CoA, subsequently decarboxylating glutaconyl-CoA to crotonyl-CoA. Deficiency of GCDH can manifest either as an isolated defect in oxidation or a combined defect affecting both oxidation and decarboxylation; however, it cannot occur as an isolated defect in decarboxylation. GCDH deficiency results in the accumulation of GA, 3-OH-GA, and, to a lesser extent, glutaconic acid and C5DC. Studies indicate that these metabolic byproducts interfere with cerebral energy metabolism [3].

GA1 is characterized by features such as macrocephaly, dystonia, gross motor developmental delay, and acute metabolic or encephalopathic crises, typically manifesting within the first three years of life following a catabolic event such as a febrile illness. These initial or subsequent crises can lead to striatal degeneration, resulting in movement disorders predominantly with dystonic or dyskinetic features. Insidious onset GA1 describes cases where striatal injury and movement disorders, such as dystonia, occur without an apparent crisis. Late-onset GA1, diagnosed after the age of six years, manifests with non-specific symptoms such as memory loss, weakness, headache, seizures, and coordination difficulties. Based on urinary glutaric acid levels, GA1 patients are classified as high excretors, having higher levels of glutaric acid in their urine, or low excretors [4]. In a study by Tamhankar et al. involving 30 children, the mean age of presentation was 10 months. The majority (over 73%) exhibited abnormal movements, 40% presented with acute encephalopathy, and 53% had an insidious disease onset [5]. Our case presented with an acute encephalitic crisis following an eight-day fever.

MRI of the brain is the preferred method for assessing GA1. Common features include enlarged subarachnoid space around the temporal poles and Sylvian fissures causing macrocephaly. Subdural hemorrhages can also be seen following minor trauma because of the rupture of bridging veins. Bilateral basal ganglia abnormalities, progressing from swelling to atrophy and necrosis, may also involve the substantia nigra, dentate nuclei, and tegmental tracts along the fourth ventricle floor in severe cases. Delayed myelination may be seen in infants [6]. In the Tamhankar et al. study of 30 patients with GA1, on MRI of the brain, 96.6% had widened Sylvian fissures, and 73.3% exhibited the bat-wing sign. Gray matter lesions were common in the caudate nucleus (50%), putamen (56.6%), and globus pallidus (60%), while white matter lesions were seen in various regions, including the periventricular white matter (26.6%) [5]. In our case, the MRI shows bifrontal and temporal atrophy with prominent sulcal spaces and Sylvian fissures. There are diffuse T2 hyperintensities in the bilateral lentiform nuclei, head of the caudate nucleus, frontal and peritrigonal white matter, dorsal pons, and floor of the fourth ventricle.

More than 200 disease-causing mutations in the *GCDH* gene have been documented, predominantly through single-base changes. Mutations such as *R227P* and *V400M* have significant enzyme activity, which leads to low or normal urinary excretion of glutaric acid. Whereas mutations such as *R402W* or *A293T* have absent or reduced enzyme activity, this results in the typical urinary metabolites. A study conducted by Radha Rama Devi et al. observed 11 different genetic mutations in 12 patients with GA1. Among these mutations, four were novel [7]. Another study on GA1 in India by Gupta et al., involving 17 patients from 15 families, identified 15 mutations, seven of which were novel [8], highlighting genetic heterogeneity in the *GCDH* gene within the Indian population.

GA, 3-OH-GA, glutaconic acid, and C5DC can be detected in body fluids (urine, plasma, and CSF) using GC/MS or TMS. Newborn screening using TMS can reveal elevated levels of C5DC. Targeted diagnostic workups for GA1 were performed in newborns with positive screening results or in cases where GA1 is suspected based on clinical and radiological signs. These tests include quantitative analysis of GA and 3-OH-GA in the urine and/or blood, molecular genetic analysis of the GCDH gene, or enzyme analysis in leukocytes or fibroblasts [8]. In our case, elevated levels of GA, 3-OH-GA, and glutaconic acid were detected through GC/MS. However, the child succumbed to illness before further diagnostic evaluation could be performed.

Management of GA1 includes parent education, dietary management, and pharmacological treatment. Up to the age of six years, a low-lysine diet is recommended, but sufficient protein intake should be ensured. After six years of age, dietary management should be age-dependent, avoiding excessive intake of high-lysine foods, and supported by regular dietary advice. Carnitine is essential for detoxifying toxic CoA compounds in GA1 by forming non-toxic, excretable C5DC. Increased glutaryl-CoA levels can deplete the intracellular CoA pool, leading to secondary carnitine depletion. Supplementing carnitine can mitigate this depletion, reduce oxidative stress, decrease the risk of striatal injury and movement disorders in early-diagnosed individuals, and lower mortality in symptomatic patients. Therefore, lifelong carnitine supplementation is recommended [9].

A study by Mhanni et al. regarding the outcome of GA1 shows that timely diagnosis and management of GA1 acute encephalopathic crises can be prevented. Patients following the dietary recommendations remain asymptomatic, whereas patients non-compliant with dietary modifications are at risk of developing severe dystonia and an encephalitic crisis [10].

## Conclusions

GA1 is a treatable form of organic aciduria. Early and prompt diagnosis, along with treatment involving dietary modification and carnitine supplementation, can prevent acute encephalitic crises and subsequent striatal injury. Early initiation of treatment also reduces the development of movement disorders such as dystonia, thereby decreasing morbidity and mortality. This case underscores the critical need for newborn screening to enable early detection and intervention.
